# Topography Mediates the Influence of Cover Crops on Soil Nitrate Levels in Row Crop Agricultural Systems

**DOI:** 10.1371/journal.pone.0143358

**Published:** 2015-11-23

**Authors:** Moslem Ladoni, Alexandra N. Kravchenko, G. Phillip Robertson

**Affiliations:** 1 The Climate Corporation, San Francisco, California, United States of America; 2 Department of Plant, Soil and Microbial Sciences, Michigan State University, East Lansing, Michigan, United States of America; 3 W.K. Kellogg Biological Station, Michigan State University, Hickory Corners, Michigan, United States of America; Chinese Academy of Sciences, CHINA

## Abstract

Supplying adequate amounts of soil N for plant growth during the growing season and across large agricultural fields is a challenge for conservational agricultural systems with cover crops. Knowledge about cover crop effects on N comes mostly from small, flat research plots and performance of cover crops across topographically diverse agricultural land is poorly understood. Our objective was to assess effects of both leguminous (red clover) and non-leguminous (winter rye) cover crops on potentially mineralizable N (PMN) and ${\text{NO}}_{3}^{-}-\text{N}$ levels across a topographically diverse landscape. We studied conventional, low-input, and organic managements in corn-soybean-wheat rotation. The rotations of low-input and organic managements included rye and red clover cover crops. The managements were implemented in twenty large undulating fields in Southwest Michigan starting from 2006. The data collection and analysis were conducted during three growing seasons of 2011, 2012 and 2013. Observational micro-plots with and without cover crops were laid within each field on three contrasting topographical positions of depression, slope and summit. Soil samples were collected 4–5 times during each growing season and analyzed for ${\text{NO}}_{3}^{-}-\text{N}$ and PMN. The results showed that all three managements were similar in their temporal and spatial distributions of NO_3_^—^N. Red clover cover crop increased ${\text{NO}}_{3}^{-}-\text{N}$ by 35% on depression, 20% on slope and 32% on summit positions. Rye cover crop had a significant 15% negative effect on ${\text{NO}}_{3}^{-}-\text{N}$ in topographical depressions but not in slope and summit positions. The magnitude of the cover crop effects on soil mineral nitrogen across topographically diverse fields was associated with the amount of cover crop growth and residue production. The results emphasize the potential environmental and economic benefits that can be generated by implementing site-specific topography-driven cover crop management in row-crop agricultural systems.

## Introduction

Nitrogen (N) is arguably the most important element for successful agricultural production and its adequate supply to plants is the key component of high yields. However, keeping plant available soil N at an adequate level across large agricultural fields during the growing season can be a challenge for agricultural management systems. Nitrogen comprises about 2% of crop biomass and is frequently deficient in crop production. Soil N exists in organic and mineral forms with the organic forms constituting 95% of soil N pools [1]. The two forms of soil mineral N absorbed by most plants are nitrate (${\text{NO}}_{3}^{-}-\text{N}$) and ammonium (${\text{NH}}_{4}^{+}-\text{N}$) [2]. In well-aerated soils during the growing season ${\text{NO}}_{3}^{-}-\text{N}$ becomes the main form of N available for crops as microbial activity quickly transforms ${\text{NH}}_{4}^{+}-\text{N}$ into ${\text{NO}}_{3}^{-}-\text{N}$ [3]. It is crucial to keep the ${\text{NO}}_{3}^{-}-\text{N}$ levels at an adequate level because, on one hand, too low levels of soil ${\text{NO}}_{3}^{-}-\text{N}$ can limit crop production and, on the other hand, too high amounts of ${\text{NO}}_{3}^{-}-\text{N}$ can lead to environmental pollution [4]. The levels of soil ${\text{NO}}_{3}^{-}-\text{N}$ vary across space and over time. Proper agricultural management needs to consider both site-specific variations as well as temporal patterns in soil ${\text{NO}}_{3}^{-}-\text{N}$ to supply optimum amounts from both organic and mineral sources.

In the US Midwest, including cover crops in the rotation grew in popularity, following raising environmental concerns about air and water pollution and soil degradation [5, 6]. Leguminous cover crops have the ability to fix atmospheric N and assimilate it into their biomass, while both legumes and non-legumes can assimilate soil ${\text{NO}}_{3}^{-}-\text{N}$ [7]. After cover crop biomass is returned to the soil, microbial mineralization transforms the organic N into mineral forms [8]. The benefits of cover crops in supplying N for main crops have been extensively studied [9–13]. However, most of the previous research was carried out in controlled, and in many cases small, experimental plots.

It is well known that the topographic variations typical of large agricultural fields can have a substantial impact on dynamics of soil mineral N as well as on performance of cover crops [14]. Soil organic matter levels vary in response to variations in topography, and the amount of organic matter is regarded as one of the main sources of ${\text{NO}}_{3}^{-}-\text{N}$ in soil [15, 16]. Corre et al. [17] suggested that spatial variations in soil organic matter, soil microbial biomass, natural drainage, plant growth, and water and nutrient redistribution caused by topography are the main factors controlling the dynamics of soil mineral N. Kay et al. [18] found that along with weather, landscape topographic patterns accounted for most of the variations in plant available N.

However, it remains unknown whether/how much the contribution of cover crops to N supply of subsequent main crops is affected by topography. Since majority of row crop agriculture is carried out in large and often topographically diverse fields, lack of such knowledge inhibits the progress in cover crop adoption by farmers. Understanding spatio-temporal patterns in cover crop N-related functioning at a scale of a typical agricultural field may resolve many of the ambiguities about cover crop performance. Such understanding is a prerequisite for development and introduction of cover crop management strategies that optimize the benefits of this promising environment conservation strategy.

The temporal variation in soil ${\text{NO}}_{3}^{-}-\text{N}$ provided by cover crops largely controls the magnitude of the benefits that cover crops can supply [19, 20]. The beneficial effects of cover crops on N will only occur if the N supplied by them becomes available during the period of high N uptake by the main crop [21]. The N in the residue of cover crops becomes available by microbial mineralization. The rate of mineralization depends largely on the C/N ratio of cover crop residues, and on environmental factors, such as soil moisture and temperature [5, 20]. Topographic variations in soil organic matter, soil moisture and soil temperature may cause differences in mineralization rates across large agricultural fields [16]. The management decisions such as timing of cover crop termination and planting of the succeeding main crop also control the synchrony between the release of mineral N from cover crop residue and the main crop N demands [22].

One of the reasons for slow progress in addressing the field scale aspects of cover crop performance are understandable difficulties associated with experimentation across topographically diverse terrain. Interactions between the cover crop presence and the topography driven factors, including spatial patterns in soil organic matter levels and/or soil erosion, make the study of their combined effects on soil N particularly difficult. For example, variations in soil organic matter across landscape lead to variations in cover crop growth, thus likely producing varied effects of cover crop on soil mineral N. On the other hand, cover crops affect surface runoff in topographically diverse fields, thus controlling redistribution of soil mineral N throughout the landscape. To produce evidence of cover crop effects free from the confounding influence of the topography associated factors requires careful and resource-consuming experimentation.

This study is based on a unique replicated field scale experiment with >20 agricultural fields with diverse topography and in corn-soybean-wheat rotation randomly assigned to three contrasting management practices, namely, conventional management, management with reduced chemical inputs and cover crops, and organic management with cover crops, with as many as 6–7 replicated fields in each management. Effect of topography on cover crop performance was addressed by sets of paired microplots strategically placed at contrasting topographical positions within each studied field. We specifically focused on potentially mineralizable N (PMN) and ${\text{NO}}_{3}^{-}-\text{N}$, as the N forms of primary relevance to plants, and monitored them during three consecutive growing seasons of 2011–2013. Our first objective was to assess temporal and topography driven variations of soil PMN and ${\text{NO}}_{3}^{-}-\text{N}$ in different agricultural managements and to identify the factors that control these variations. Our second objective was to assess the particular contribution of cover crops to PMN and ${\text{NO}}_{3}^{-}-\text{N}$ levels across topographically diverse terrain during the growing season.

## Materials and Methods

### Ethics Statement

Permission for collecting soil samples has been obtained from the Executive Committee of the Long Term Ecological Research site experiment at W. K. Kellogg Biological Station (LTER KBS). Permission was obtained upon submission of the site use request form (http://lter.kbs.msu.edu/research/conducting-research/). The samples did not involve any endangered species.

### Fields and Treatments Set-Up

The study was conducted at the Scale-up Experiment established in 2006 at LTER KBS in southwest Michigan (42° 24' N, 85° 24' W) (Robertson and Hamilton 2015). The entire experiment consists of 27 agricultural fields, ranging in size from 3.1 to 7.9 ha. The dominant soil series are Kalamazoo (fine-loamy, mixed, mesic Typic Hapludalfs) and Oshtemo (coarse-loamy, mixed, mesic Typic Hapludalfs).

Three agricultural management practices were established in 2006: conventional, low-input and organic management. The studied fields were in corn-soybean-wheat rotation, and every year all three phases of the rotation were present in all three studied management practices. The overall experimental design was a randomized complete block with three replications of each management practice in each main crop. That is, a total of 9 fields were assigned to each management practice; and each year corn, soybean and wheat were planted each in 3 of these fields. Since primary focus of our study was the effects of topography we worked with only 20 of the experimental fields, namely with the fields with sufficient topographical diversity.

The rotations of low-input and organic management treatments included two cover crops: a leguminous cover crop, red clover (*Trifolium pratense* L.), and cereal rye (*Secale cereale*), a non-leguminous cover crop. Red clover was frost-seeded in winter wheat in March and then plowed into the soil prior to corn planting in May of the following year. Rye was planted after the corn harvest, in October, and then plowed into the soil prior to soybean planting in May of the following year.

The conventional management received N and K fertilizers and necessary pesticides at the rates recommended by Michigan State University Extension and soil tests[23]. All of the pesticide, K fertilizer and a third of the N fertilizer were applied by broadcasting in spring before corn planting. The remaining N fertilizer was applied banded within rows approximately one month after planting. The low-input management received 1/3 of the N fertilizer and herbicide amounts applied to the conventional systems; herbicides were banded at full strength only in rows rather than broadcast. N fertilizer was broadcast in spring before planting corn. The organic system did not receive any synthetic chemical inputs.

The primary tillage in all three management practices was spring chisel plowing. The secondary tillage consisted of disking before wheat planting, field conditioning with a soil finisher before soybean and corn planting, and inter-row cultivation for soybean and corn. The organic systems received additional inter-row cultivation and rotary hoeing as needed for weed control. Details of the management related activities (i.e. tillage, planting, harvesting, etc.) can be found at http://lter.kbs.msu.edu/protocols/104 (verified 26 July 2015).

**Fig 1:**
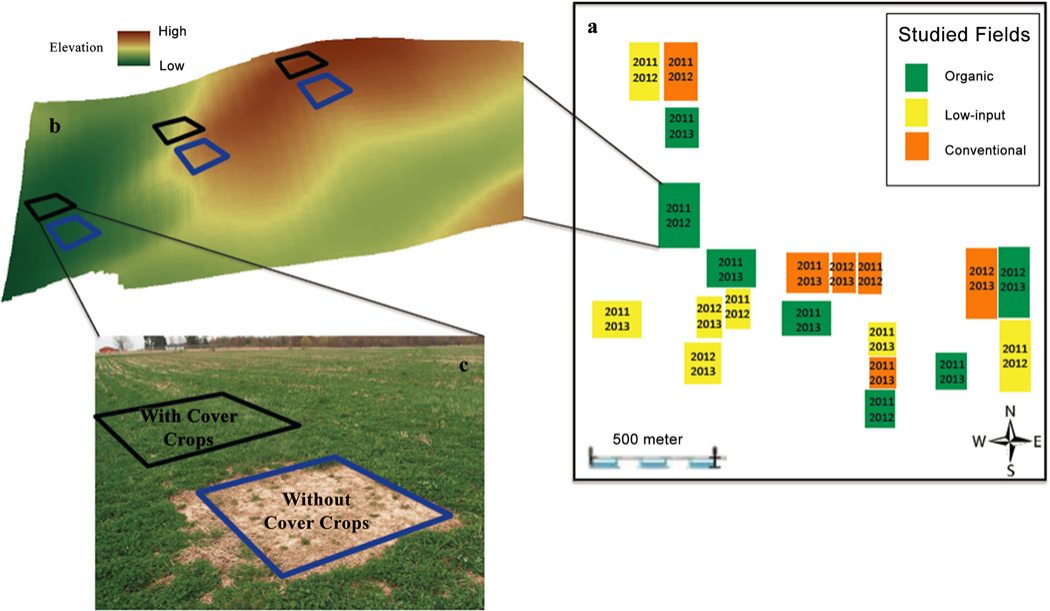
The experimental lay-out: a) the study area with locations of the monitored fields from the three studied treatments and the years when each field was sampled; b) a schematic presentation of a topographical transect in one of the studied fields; and c) 4×4 m cover and bare micro-plots within a selected topographical position.

Data were collected in 2011, 2012 and 2013. Note that we only sampled the fields that were in corn and soybean phases of the rotation. Thus the 20 studied fields were sampled twice during the 2011–2013 period and the years when each field was sampled are shown on Fig 1. Overall, in 2011, we sampled 4 fields from conventional management, 5 fields from low-input management, and 5 fields from organic management. In 2012, 4 fields from conventional management, 4 fields from low-input management, and 4 fields from organic management were sampled. In 2013, we sampled 4, 5, and 3 fields from conventional, low-input, and organic managements, respectively.

Topographical attributes of the fields were derived from the digital elevation model of the area at 2 m resolution. The attributes included relative elevation, terrain slope, flow length, and flow accumulation. Linear discriminant analysis was applied to the topographical attributes to classify the studied fields into three topographical positions: summit, slope and depression as described by Munoz et al. [24].

Within each field we identified topographical transects across summit, slope, and depression positions. The number of transects depended on the size of the field and the topographic variability within the field. In larger, more undulating fields up to four transects were identified whereas small and relatively flat fields typically had only one transect. For the fields under cover crop management along each transect in each of the three topographical positions we laid out two adjacent 4 x 4 m micro-plots. One of the micro-plots had the cover crops similar to the entire field (“cover”), while the other was kept bare of weeds and cover crop plants by herbicide applied after plant emergence, i.e., in summer after wheat harvest for fields in red clover and in early spring for fields with rye (“bare”) (Fig 1). The micro-plots were established in 2010 and then continuously maintained in the same locations for the entire duration of the study. In conventional management fields, which did not have cover crop in the rotation, we established only bare micro-plots. Soil and cover crop biomass was collected from micro-plots as described below.

### Sample Collection and Analysis

We sampled biomass of rye and red clover from all cover crop micro-plots in spring 2–3 days before their termination. For biomass sampling a 0.5 by 0.5 m square was randomly placed in a cover micro-plot and the total aboveground cover crop biomass was collected. The biomass sample was dried and weighed after drying.

Biomass of corn and soybean was sampled from cover micro-plots in low-input and organic fields and from bare micro-plot in conventional fields at the harvest times in Fall 2010, 2011 and 2012. The total biomass was measured along two rows of 1 m length. The biomass samples were dried for 78 hours at 70^°^C and weighed after drying.

Soil samples were collected from 0–15 cm depth from micro-plots four times during each growing season using a push probe (2 cm diameter). The first soil sampling was conducted in spring 2–3 days before cover crop termination. The second sampling was conducted two weeks after plowing, the third sampling was conducted 8 weeks after plowing and the last sampling was conducted after harvesting the main crop. After each sampling the soil samples were air-dried for 78 hours and then sieved to 2 mm.

We measured soil organic nitrogen (SON) once at the beginning of the study in all of the fields. The measurements were made for cover micro-plots in low-input and organic fields and bare micro-plot in conventional fields. SON was measured via dry combustion method using a Costech ECS 4010 CHNSO analyzer.

Soil ${\text{NO}}_{3}^{-}-\text{N}$ was measured in all of the collected samples. For that, 10 g of air-dried soil was shaken with 50 ml of 1M KCl for 30 minutes. The mixture was then extracted through paper filter. ${\text{NO}}_{3}^{-}-\text{N}$ content of filtered extracts was determined using the LAChat rapid flow injection unit [25].

Potential mineralizable N (PMN) was measured as per Robertson et al. [26] at three time points during the growing season, i.e., before plowing, eight weeks after plowing, and after harvest. For that, 10 g of soil was extracted as described previously and the amounts of ${\text{NO}}_{3}^{-}-\text{N}$ and ${\text{NH}}_{4}^{+}-\text{N}$ were measured in the extract. Another 10 g of air-dried soil was brought to 50% water holding capacity moisture level and was incubated in a 25^°^C incubator for 14 days. The sample was then extracted in a similar way as described above and soil ${\text{NO}}_{3}^{-}-\text{N}$ and ${\text{NH}}_{4}^{+}-\text{N}$ was measured. PMN was calculated as the difference between the total amounts of mineral N (${\text{NO}}_{3}^{-}-\text{N}$ and ${\text{NH}}_{4}^{+}-\text{N}$) in the initial and after-incubation measurements.

### Statistical Analysis

Two sets of data analyses were conducted using the MIXED procedure of SAS 9.2 (Cary, NC). In the first set of analyses our purpose was to compare the conventional and cover crop based managements at different topographical positions. For this analysis we used data from the micro-plots in conventional management fields and from the cover micro-plots in the cover crop based management fields. The statistical model for this analysis included agricultural management, topographical position, sampling date and the interactions between them as the fixed factors with date treated as a repeated measure. Fields nested in agricultural managements were included in the model as the random factor and were used as an error term for testing the agricultural management effect.

In the second set of data analyses our purpose was to directly address the effects of cover crop presence at different topographical positions. For this analysis we used the data from the bare and cover micro-plots from the low-input and organic fields. The statistical model included topographical position, cover crop presence, sampling date and the interactions between them as the fixed factors with date treated as a repeated measure. Fields and interaction between fields and topographical positions were included in the model as random factors. Field was used as a blocking factor while field by topography interaction was used as an error term for testing the effect of topography. Normality of the residuals and homogeneity of variance assumptions were checked for all studied variables in both sets of data analyses. Means were compared when the respective main and/or interaction effects were statistically significant at the p<0.05 level. The differences among the treatment means were reported as statistically significant at p<0.05 level and also reported as trends when statistically significant at p<0.1 level. For both data analyses the numbers of samples >150, as it was based on 14–20 studied fields with 1–3 transects per field, with 3 topographical positions per transect, and with two micro-plots at each position of the cover crop-based fields.

Three simple linear regression analyses were conducted to examine influences of SON and cover crop biomass on soil ${\text{NO}}_{3}^{-}-\text{N}$ and PMN. The first regression analysis was conducted with SON as the independent variable for soil ${\text{NO}}_{3}^{-}-\text{N}$ and PMN as the dependent variables. In the second regression analysis we looked at the difference in ${\text{NO}}_{3}^{-}-\text{N}$ between cover and bare micro-plots at each topographical position as the dependent variable and at cover crop biomass as the independent variable. The third analysis was conducted with the difference in PMN between cover and bare micro-plots as the dependent variable and the cover crop biomass as the independent variable. Using the difference between cover and bare members of each micro-plot pair enabled us to isolate the effect of cover crop presence from confounding influences of other topography and soil driven factors, including spatial patterns in soil organic matter and surface runoff. All regression analyses were conducted using the MIXED procedure of SAS, which enabled us to include fields nested within year in the random statement of the regression analyses.

## Results

### Topographic and Management Effects on the Studied Plant and Soil Characteristics

The largest corn and soybean biomass was observed in depression areas under all three managements (Table 1). The smallest biomass of corn and soybean was observed in slopes under all managements (p<0.05). Numerically, the differences in corn and soybean biomass between slopes and depressions were the largest under organic management compared to the conventional and low-input managements.

**Table 1:** Means and standard errors (in parentheses) of ${\text{NO}}_{3}^{-}-\text{N}$, SON (soil organic nitrogen), fertilizer inputs, and biomass of red clover, rye, corn and soybean in the studied managements across the studied 20 fields. Data are averages from 2011–2013.

	Conventional			Low-input			Organic		
	Depression	Slope	Summit	Depression	Slope	Summit	Depression	Slope	Summit
${\text{NO}}_{3}^{-}-\text{N}$, mg kg^-1^	7.8 (1.6) a*	4.2 (1.5) b	7.5 (1.5) a	6.7 (1.4) a	3.3 (1.4) b	4.1 (1.4) b	9.4 (1.4) a	4.5 (1.4) b	6.4 (1.4) b
SON, mg kg^-1^	0.53 (0.03) a	0.44 (0.03) b	0.48 (0.03) ab	0.53 (0.03) a	0.40 (0.03) b	0.44 (0.03) b	0.44 (0.03) a	0.44 (0.03) a	0.44 (0.03) a
Fertilizer, kg ha^-1^	120	120	120	40	40	40	0	0	0
Red clover biomass, kg ha^-1^	N/A	N/A	N/A	1761 (278)a	1162(278) b	1521 (278) ab	1653 (284) a	1482 (284) a	1617 (284) a
Rye biomass, kg ha^-1^	N/A	N/A	N/A	633 (87) a	329 (87) b	296 (87) b	769 (77) a	496 (77) b	513 (77) b
Corn biomass, kg ha^-1^	18600 (1100) a	13700 (1100) b	17900 (1100) a	15800 (1100) a	11100 (1100) b	11900 (1100) b	15000 (1300) a	7600 (1300) b	10000 (1300) b
Soybean biomass, kg ha^-1^	11664 (490) a	6426 (490) b	6954 (490) b	10033 (480) a	6265(480) b	8900 (480) a	9263 (510) a	4650 (510) b	5900 (510) b

* Different lowercase letters in each row within each management mark significant differences among the topographical positions (p<0.05).

SON was the greatest in depressions compared to slopes under conventional and low-input managements. We did not observe a significant difference in SON between topographical positions under organic management (p<0.05). The regression analysis showed a significant relationship of SON with ${\text{NO}}_{3}^{-}-\text{N}$ and PMN under all three managements (p<0.05). The regression slopes of SON with PMN were the largest under organic management (Table 2).

**Table 2:** The relationships between ${\text{NO}}_{3}^{-}-\text{N}$, PMN (potential mineralizable N) and SON (soil organic nitrogen). Shown are regression slopes with standard errors (in parenthesis).

Dependent variable mg kg^-1^	Independent variable mg kg^-1^	Management		
		Conventional	Low-input	Organic
${\text{NO}}_{3}^{-}-\text{N}$	SON	11.6 (4.5) a*	10.6 (3.9) a	17.3 (4.7) a
PMN	SON	21.8 (5.3) b	25.3 (4.7) b	42.7 (6.5) a

*All of the regression slopes were significantly different from zero (p<0.05). Within each row different lowercase letters indicate significant differences among the managements (p<0.05).

Cover crop biomass was typically the largest in depressions followed by summits and slopes. Red clover aboveground biomass inputs were larger compared to rye (Table 1).

Managements had no significant effect on soil ${\text{NO}}_{3}^{-}-\text{N}$ levels. However, the influence of topography on ${\text{NO}}_{3}^{-}-\text{N}$ was statistically significant (p<0.05). Under all three managements the slopes had lower ${\text{NO}}_{3}^{-}-\text{N}$ compared to depression areas (Table 1). Under low-input and organic management topographical summits also had significantly lower ${\text{NO}}_{3}^{-}-\text{N}$ compared to depressions but under conventional management there was no significant difference between depressions and summits (p<0.05).

### The Effects of Cover Crops on Soil PMN and NO^-^_3_-N at Different Topographical Positions

**Fig 2:**
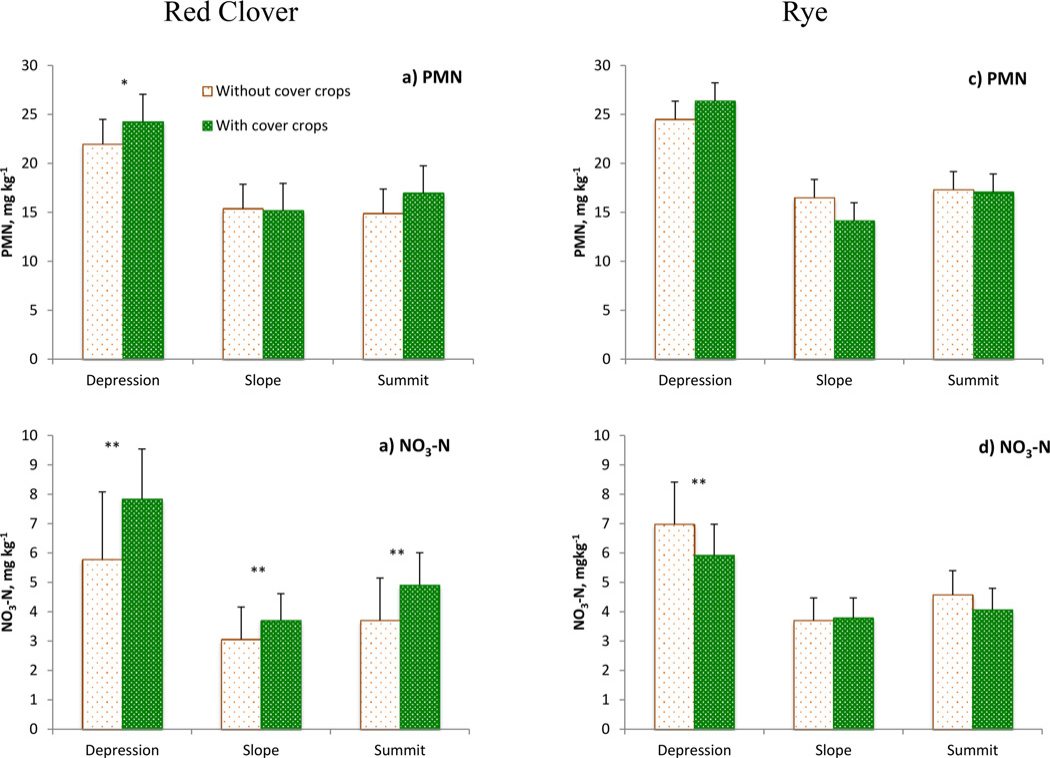
Soil potential mineralizable N (PMN) and ${\text{NO}}_{3}^{-}-\text{N}$ in bare and cover micro-plots of the studied topographical positions following red clover and rye cover crops. The data shown are the averages from 2011, 2012 and 2013 results. Error bars represent standard errors. The ** and ^*^ mark the cases when the differences between cover and bare micro-plots were statistically significant at p<0.05 and p<0.1, respectively.

There was a trend of higher PMN in cover crop micro-plots as compared to bare micro-plots in depression areas which was significant at p<0.1 under red clover (Fig 2A), but not significant under rye (Fig 2C). No significant differences between cover and bare micro-plots in terms of PMN were observed at other topographical positions.

Soil ${\text{NO}}_{3}^{-}-\text{N}$ increased significantly due to the presence of red clover in all topographical positions (Fig 2B). The presence of rye tended to have an opposite effect on soil ${\text{NO}}_{3}^{-}-\text{N}$. It significantly decreased ${\text{NO}}_{3}^{-}-\text{N}$ in depressions, while no significant differences between bare and cover micro-plots were observed in slopes and summits (Fig 2D).

We used regression analysis to assess the relationship between cover crop biomass and its effect on soil ${\text{NO}}_{3}^{-}-\text{N}$, expressed as the difference in ${\text{NO}}_{3}^{-}-\text{N}$ between cover and bare micro-plots (Table 3). Regression analysis showed that the aboveground biomass of red clover was positively related to the ${\text{NO}}_{3}^{-}-\text{N}$ difference between cover and bare micro-plots in depressions and summits (p<0.05). A negative relationship between the aboveground biomass of rye and soil ${\text{NO}}_{3}^{-}-\text{N}$ was observed in depressions (p<0.05). Only weak positive relationship (p<0.1) was observed between red clover biomass and the cover-bare difference in terms of PMN in depressions, but not in other topographical positions.

**Table 3:** The relationships between the differences between cover and bare micro-plots in terms of ${\text{NO}}_{3}^{-}-\text{N}$ and PMN (potential mineralizable N) and the cover crop biomass.

Dependent variable mg kg^-1^	Independent variable	Position	Regression slope/ Std error
${\mathrm{NO}}_{3}^{-}-N$ **difference between cover and bare**	**Red clover biomass, kg ha** ^**-1**^	Depression	0.0015/0.0007^**^
		Slope	0.0010/0.0006 ^NS^
		Summit	0.0013/0.0005^**^
	**Rye biomass, kg ha** ^**-1**^	Depression	-0.0028/0.001^**^
		Slope	-0.0014/0.002^NS^
		Summit	-0.0001/0.002^NS^
**PMN difference between cover and bare**	**Red clover biomass, kg ha** ^**-1**^	Depression	0.0022/0.001^*^
			0.0004/0.001^NS^
		Summit	0.0001/0.001^NS^
	**Rye biomass, kg ha** ^**-1**^	Depression	-0.002/0.003^NS^
		Slope	0.007/0.005^NS^
		Summit	-0.005/0.0006^NS^

The ** and * marks the cases where the relationships between the studied variables were statistically significant at p<0.05 and p<0.1 levels, respectively. NS: not significant at p<0.1.

### The Effects of Cover Crops on Temporal Patterns of Soil PMN and NO^-^_3_-N

**Fig 3:**
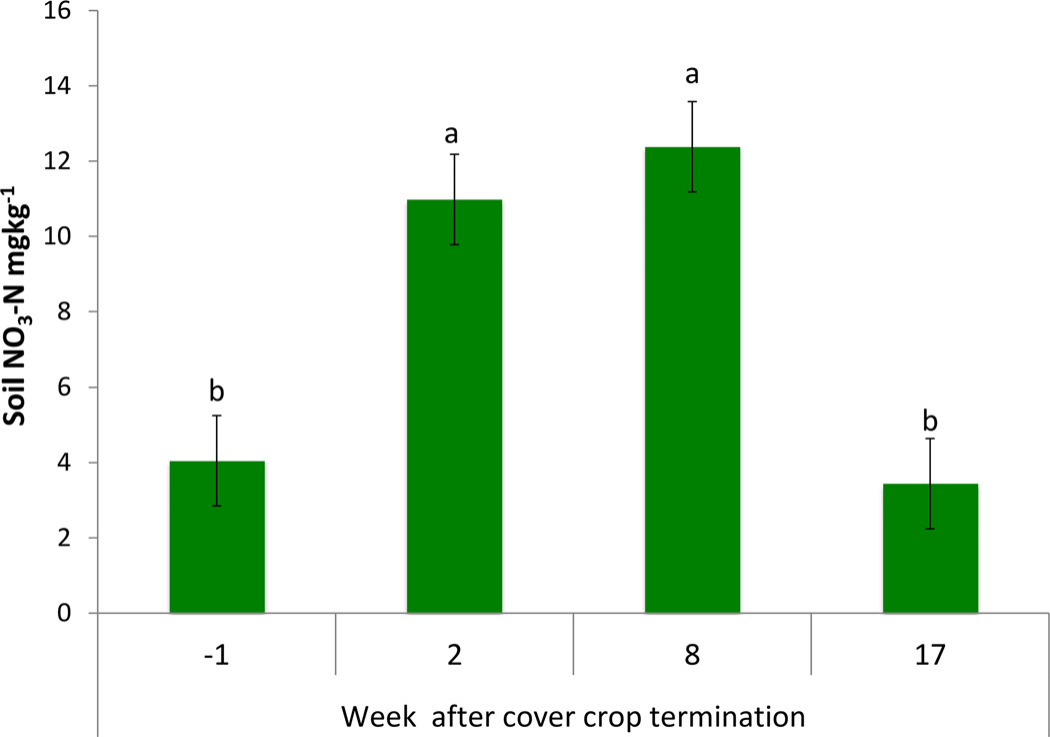
${\text{NO}}_{3}^{-}-\text{N}$ in soil at different sampling dates during growing season. The data shown are the averages from 2011, 2012 and 2013 results of all three studied treatments. Error bars represent standard errors. Different letters above the bars indicate significant differences among sampling dates (p < 0.05).

The largest ${\text{NO}}_{3}^{-}-\text{N}$ was observed in soil two to eight weeks after plowing in all three managements (p<0.05) and then it decreased in after-harvest measurements (Fig 3).

**Fig 4:**
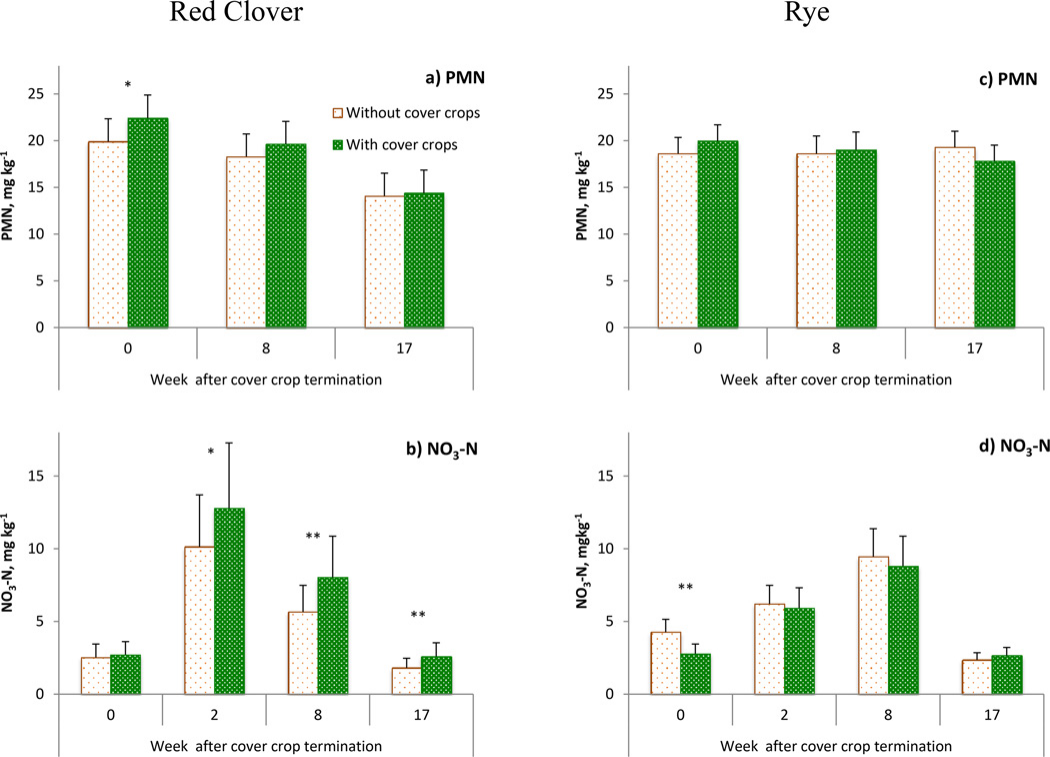
${\text{NO}}_{3}^{-}-\text{N}$ and potential mineralizable N (PMN) in bare and cover micro-plots at the studied sampling times following red clover (a and b) and rye (c and d) cover crops. The data shown are the averages from 2011, 2012 and 2013 results. Error bars represent standard errors. The ** and ^*^ mark the cases when the differences between cover and bare micro-plots were statistically significant at p<0.05 and p<0.1, respectively.

Across all studied topographical positions the presence of red clover increased PMN at the sampling conducted shortly before cover crop termination (p<0.1) (Fig 4A). The positive effects of red clover on PMN decreased later in the growing season, and eight weeks after plowing no significant difference between bare and cover micro-plots in PMN was observed (Fig 4A). However, a positive effect of red clover presence on ${\text{NO}}_{3}^{-}-\text{N}$ appeared two weeks after red clover termination (p<0.1) and persisted for at least eight weeks after plowing and even after the main crop harvest (p<0.05).

The effects of rye’s presence on ${\text{NO}}_{3}^{-}-\text{N}$ was opposite to that of red clover. Rye did not have a significant effect on PMN before its termination (Fig 4C); however, soil ${\text{NO}}_{3}^{-}-\text{N}$ decreased under the soil covered with rye compared to bare soil (Fig 4D). The negative effects did not last, as two weeks after termination of rye the ${\text{NO}}_{3}^{-}-\text{N}$ levels in cover and bare micro-plots were not significantly different from each other (p<0.1) (Fig 4D).

## Discussion

The main two hypotheses of our study were (i) that the magnitudes of the effects from cover crops on soil PMN and ${\text{NO}}_{3}^{-}-\text{N}$ vary across topographical gradients, and (ii) that the amounts of biomass produced by cover crops are one of the drivers for the size of the cover crop effects. Both hypotheses were supported by the data. For both studied cover crops, i.e., leguminous red clover and non-leguminous rye, the greatest influence of cover crop presence in terms of both PMN and ${\text{NO}}_{3}^{-}-\text{N}$ was observed at topographical depressions.

### Spatial Distribution of Soil NO^-^_3_-N under Different Managements

The spatial distribution of ${\text{NO}}_{3}^{-}-\text{N}$ observed under the three studied managements (Table 1) was the outcome of a balance between inputs and outputs of mineral N controlled mainly by: spatial distribution patterns in SON mineralization, N fertilizer application, uptake by the main crop, and uptake/input from cover crops. While our results showed that the combined effect of all contributors to soil ${\text{NO}}_{3}^{-}-\text{N}$ resulted in its similar spatial and temporal patterns in all three studied managements, to fully appreciate the landscape scale environmental and ecological effects of cover crop presence, it is important to understand the specific contribution of each ${\text{NO}}_{3}^{-}-\text{N}$ source/sink to the spatial and temporal distribution patterns of ${\text{NO}}_{3}^{-}-\text{N}$.

Soil organic N as one of the main sources of soil ${\text{NO}}_{3}^{-}-\text{N}$ is likely the main cause for the observed spatial patterns in ${\text{NO}}_{3}^{-}-\text{N}$ distributions. The regression analysis showed that across all years and managements, for every 1 mg kg^-1^ increase in SON, soil ${\text{NO}}_{3}^{-}-\text{N}$ increased as much as 11.6, 10.6 and 17.3 mg kg^-1^ in conventional, low-input and organic managements, respectively (Table 2). These results are consistent with previous studies by Garten and Ashwood [27], Soon and Malhi [28], Dharmakeerthi et al. [16] and Corre et al. [17] who reported soil organic matter as the main factor controlling the spatial distribution of ${\text{NO}}_{3}^{-}-\text{N}$. It should be noted that in the organic management, where no N fertilizer is applied, the contribution of SON to ${\text{NO}}_{3}^{-}-\text{N}$ tended to be larger compared to low-input and conventional managements.

Fertilizer was another main source of input in the conventional and low-input fields. However, its application should have had only a relatively minor influence on the variations in ${\text{NO}}_{3}^{-}-\text{N}$ across topography because fertilizers were uniformly applied through entire fields. Nevertheless, spatial redistribution along the topographic gradients via surface run-off could have depleted some of the applied mineral N from slopes and accumulated it in depressions.

The largest biomass of corn and soybean in depressions indicated a larger plant uptake of ${\text{NO}}_{3}^{-}-\text{N}$ there as compared to terrain slopes, which probably contributed to a reduction in the spatial variability of ${\text{NO}}_{3}^{-}-\text{N}$. The effects of topography on the growth and thus biomass of main crops are well-known and our results are consistent with a multitude of previous reports indicating that topographical depressions tend to provide optimal growth conditions for Midwest corn and soybean crops (e.g., [14, 29]). A larger magnitude of differences between depressions and slopes in terms of corn biomass was observed in the organic fields as compared to conventional and low-input managements. In both low-input and conventional managements corn produced 30% less biomass on slopes as compared to depressions; while under organic management slopes produced 50% less corn biomass than depressions. Our results are in agreement with those of Kravchenko et al. [30] who observed the largest spatial variability in terms of crop grain yield under organic management compared to low-input and conventional managements.

Both cover crops produced larger biomass in depression areas (Table 1). Similar to the main crops, the most likely reason for the observed result was better growing conditions in the depressions, driven by higher soil organic matter and better soil structure as compared to slopes and summits [14].

### The Effects of Cover Crops on Spatial Distribution of Soil PMN and NO^-^_3_-N

The discussed above multiple and often counter-acting sources of variation present across large fields substantially hinder success in assessing field-scale effects of cover crops on soil N. Our micro-plot design enabled us to address the cover crop effects across large topographically and edaphically diverse fields with minimum interference from other sources of variability. Having a set of two micro-plots with and without cover crops that were laid out in close proximity to each other enabled us to examine the effects of cover crop presence under similar soil properties and plant growth conditions. Note that since the results for cover crop biomass, ${\text{NO}}_{3}^{-}-\text{N}$, and PMN in organic and low input fields were very similar, for the discussions of cover crop influences we combined the data from these two managements.

Better cover crop growth and subsequent larger residue inputs in depressions led to greater PMN levels in cover as compared to bare micro-plots (Fig 2A and 2C). The microbial decomposition of cover crops residue releases mineral N into soil and thus enhances soil PMN, especially under red clover due to its high N levels. Cover crop residue inputs on slopes apparently were not sufficient to notably increase PMN in the cover micro-plots. A larger effect of cover crop presence on PMN for a legume red clover (Fig 2A) as compared to a non-legume rye (Fig 2C) reflects both extra N inputs and longer duration of the red clover cover. This result is consistent with the report of Schomberg and Endale [13], who observed larger contribution from crimson clover to soil mineralizable N compared to rye under cotton production system.

Our observations of positive effects on soil ${\text{NO}}_{3}^{-}-\text{N}$ from leguminous cover crop (Fig 2B) as opposed to negative effects from non-leguminous cover crop (Fig 2D) were consistent with multiple previous reports (e.g. [12, 21, 31–33]). However, we would like to particularly emphasize the importance of significant positive effects from red clover presence on terrain slopes. Low soil productivity and often deficient N on slopes of these undulating fields is a common cause for the overall lower main crop yields. The observed increase in soil ${\text{NO}}_{3}^{-}-\text{N}$ on slopes suggests that legume cover cropping can be a solution for improving soil productivity in those parts of the landscape where an improvement is particularly needed.

The effect of rye cover presence on ${\text{NO}}_{3}^{-}-\text{N}$ varied depending on topographical position (Fig 2D), being significantly negative in depressions, while non-existing on slopes. Since topographical depressions are particularly vulnerable to ${\text{NO}}_{3}^{-}-\text{N}$ leaching to groundwater, the observed decreases in ${\text{NO}}_{3}^{-}-\text{N}$ in depressions due to rye cover presence point to the areas on a landscape where utilization of a non-legume N-scavenging cover crop can produce the most substantial environmental benefits.

The reasons behind the observed lack of negative effect of rye on slopes are not fully understood. The low biomass of rye on terrain slopes as compared to more fertile depressions could be one of the contributing factors. However, comparisons between cover and bare micro-plots in terms of different fractions of soil organic matter indicated that, despite the low biomass production, the presence of rye cover crop did have a beneficial effect on soil organic matter on slopes [34]. Combined findings of this study and Ladoni et al. [34] suggest that planting rye cover crop on eroded unfertile slopes leads to a win-win situation: the negative effects of rye on soil ${\text{NO}}_{3}^{-}-\text{N}$ there are negligible while positive effects of rye on soil organic matter are present and likely to increase with time.

Consistent with our hypothesis, the amount of aboveground red clover biomass returned to soil was a factor influencing the magnitude of the effects that the cover crop presence had on soil ${\text{NO}}_{3}^{-}-\text{N}$ (Table 3). However, the results also indicated that below ground contribution from red clover roots and root exudates, unaccounted for in our study, also might have been substantial. For example, from the analysis of the relationship between the increase in ${\text{NO}}_{3}^{-}-\text{N}$ due to cover crop presence and the red clover aboveground biomass in depressions, we can expect that for each 1000 kg ha^-1^ increase in biomass an approximately 1.5 mg kg^-1^ increase in ${\text{NO}}_{3}^{-}-\text{N}$ takes place (Table 3). However, with the average red clover biomass values of only 600–700 kg ha^-1^ observed in our study (Table 1), the actual observed difference between bare and cover micro-plots in terms of soil ${\text{NO}}_{3}^{-}-\text{N}$ was >2 mg kg^-1^ (Fig 2B). Thus, the aboveground biomass of red clover was responsible only for a part of the differences in ${\text{NO}}_{3}^{-}-\text{N}$ between cover and bare micro-plots. Schomber and Endale [13] showed that red clover enriched soil N via root activities during its growth, while its N-rich residue contributed to soil N after its termination. Andraski and Bundy [35] observed that the presence of cover crops enriched soil N even when the aboveground biomass of cover crop was harvested and concluded that roots of cover crops increased soil mineral N.

The magnitude of the negative effect of rye presence on soil ${\text{NO}}_{3}^{-}-\text{N}$ was also related to the rye biomass, however, the relationship was statistically significant only in depressions. In depressions, the regression analysis showed that per every 1000 kg ha^-1^ of aboveground rye biomass a 2.8 mg kg^-1^ decrease in ${\text{NO}}_{3}^{-}-\text{N}$ occurred.

### The Effects of Cover Crops on Temporal Distribution of Soil PMN and NO^-^_3_-N

The temporal patterns observed in our study are consistent with previous reports [31, 36](Fig 3). The ${\text{NO}}_{3}^{-}-\text{N}$ increased two weeks after plowing reflecting (i) the release of mineral N from mineralization of soil organic matter in all three studied treatments, (ii) the decomposition of cover crop residue in low-input and organic managements, and (iii) the nitrification of the applied fertilizer in conventional and low-input managements. Higher ${\text{NO}}_{3}^{-}-\text{N}$ values persisted until eight weeks after plowing when plant uptake of ${\text{NO}}_{3}^{-}-\text{N}$ increased and soil ${\text{NO}}_{3}^{-}-\text{N}$ declined (Fig 3). Surprisingly, we observed no significant differences among the three studied managements during the three studied growing seasons in terms of ${\text{NO}}_{3}^{-}-\text{N}$ levels (Table 1).

Greater PMN prior to red clover termination suggested that it increased the pools of soil N even before its aboveground biomass was returned to soil, most likely via root exudation of labile C- and N-rich compounds (Fig 4A). However, it is only after cover crop termination that the increased PMN levels started to materialize into higher ${\text{NO}}_{3}^{-}-\text{N}$ (Fig 4B). Prior to red clover termination the cover plots had higher PMN but similar ${\text{NO}}_{3}^{-}-\text{N}$ levels to those of bare micro-plots. The low temperatures and resulting low microbial mineralization of early spring could be one reason that the organic N released by red clover roots was not immediately transformed into ${\text{NO}}_{3}^{-}-\text{N}$.

Fast decomposition of N-rich residue of red clover in soil is likely the reason for smaller effects of red clover on PMN later in the growing season (Fig 4A). Our assessment of residue decomposition using litter bags showed that more than 95% of red clover residue decomposed during the two months after cover crop termination [34]. Rapid decomposition of legume cover crop residue was also reported by Schomberg and Endale [13] and Starovoytov et al. [31]. After assessing the mineralization of cover crop residue, Schomberg and Endale [13] concluded that only a small portion of N from cover crops becomes available for the subsequent crop. Nevertheless, our results indicated that presence of red clover had positive effects on ${\text{NO}}_{3}^{-}-\text{N}$ and that those effects started early in the season and lasted almost entire growing season.

The decrease in ${\text{NO}}_{3}^{-}-\text{N}$ early in the season revealed the ability of rye to scavenge the soil ${\text{NO}}_{3}^{-}-\text{N}$, as frequently reported in the literature [5, 6]. Wortman et al. [20] observed that negative effects from a mixture of cover crops on soil ${\text{NO}}_{3}^{-}-\text{N}$ lasted until 81 days after termination. The shorter negative effects in our study could be related to a leguminous main crop, i.e., soybean, shortly following after rye termination. Schomberg and Endale [13] and Lawson et al. [37] suggested that rye can assimilate ${\text{NO}}_{3}^{-}-\text{N}$ early in the growing season and then release it to soil later producing close synchrony with the main crop plant needs. Our results also showed an assimilation of ${\text{NO}}_{3}^{-}-\text{N}$ early in the season, however, we do not have evidence that mineralization of rye residue increased the ${\text{NO}}_{3}^{-}-\text{N}$ levels in soil later in the growing season.

### Management Implications

Our study highlights the two items that could be of specific relevance to cover cropping implementation and management in fields of undulating terrain. The first item is potential environmental and economic benefits of site-specific topography-driven cover crop management. Management decisions regarding where to plant cover crops can vary depending on the management goals and complexity of the terrain. For example, cover crops seem to be particularly advantageous on eroded unfertile slopes where legumes bring the needed N inputs, and rye does not result in substantial N reductions, while all cover crops contribute to erosion control and C sequestration there. On the other hand, if N leaching is the major concern, low located depression areas is where rye cover crop can be particularly advantageous for scavenging ${\text{NO}}_{3}^{-}-\text{N}$.

The second item is the importance of not only above ground, but also belowground inputs from cover crop plants. For example, the aboveground biomass of red clover explained only slightly more than a half of the observed increases in soil ${\text{NO}}_{3}^{-}-\text{N}$. This suggests that root activity and belowground residue are as important as the aboveground contributions. This observation points to extra opportunities for cover crop use, e.g., farmers can still derive soil N- and C-related benefits from their cover crops even if the aboveground biomass is harvested or grazed.

## Conclusions

1- Despite differences in cover cropping and fertilizer application practices, similar spatial and temporal patterns in soil ${\text{NO}}_{3}^{-}-\text{N}$ were observed in all three managements, including conventional management that received synthetic N fertilization as well as organic management with no fertilizer inputs. Soil organic N was similarly distributed under all managements and was perhaps the main factor controlling spatial distribution of soil ${\text{NO}}_{3}^{-}-\text{N}$ in these large topographically and edaphically diverse fields.

2- The presence of red clover had a positive effect on soil ${\text{NO}}_{3}^{-}-\text{N}$ across all topographical positions. Including red clover in rotation would have benefits in enhancing ${\text{NO}}_{3}^{-}-\text{N}$ availability, particularly on the eroded topographical slopes where low soil quality results in poor performance of main crops. The increases in ${\text{NO}}_{3}^{-}-\text{N}$ due to red clover presence were positively associated with the amounts of aboveground red clover biomass returned to the soil prior to corn planting. However, the actual observed increases in ${\text{NO}}_{3}^{-}-\text{N}$ were greater than those predicted by associations with the aboveground biomass, pointing to the unaccounted positive contributions of the belowground inputs.

3- The effects of rye presence varied depending on topography. Presence of rye significantly decreased soil ${\text{NO}}_{3}^{-}-\text{N}$ in depressions, however it had no significant effect on ${\text{NO}}_{3}^{-}-\text{N}$ summits and slopes. Lower rye biomass on slopes and summits along with lower microbial activity reported at these topographical positions are possible reasons for the observed differences.

4- Positive influence of red clover presence on soil ${\text{NO}}_{3}^{-}-\text{N}$ continued throughout the entire growing season. Apparently, the lack of the synchrony of release of N from red clover residue and the plant needs early in the season was not a concern for the fields in our study. Rye had the ability to scavenge soil ${\text{NO}}_{3}^{-}-\text{N}$ before its termination, however, soil ${\text{NO}}_{3}^{-}-\text{N}$ gradually increased after termination of rye.
